# Supplementary material for: Myo5b Transports Fibronectin-Containing Vesicles and Facilitates FN1 Secretion from Human Pleural Mesothelial Cells
Source: Int J Mol Sci. 2022 Apr 27;23(9):4823. doi: 10.3390/ijms23094823 (PMC9101030; doi:10.3390/ijms23094823)
Supplement: Supplementary file 1 [file ijms-23-04823-s001.zip › Supplementary Materials.pdf]

Supplementary Materials

Myo5b Transports Fibronectin-Containing Vesicles and Facilitates FN1 Secretion from Human Pleural Mesothelial Cells

Tsuyoshi Sakai, Young-yeon Choo, Osamu Sato, Reiko Ikebe, Ann Jeffers, Steven Idell, Torry Tucker and Mitsuo Ikebe *

Department of Cellular and Molecular Biology, The University of Texas Health Science Center at Tyler, 11937 US Highway 271, Tyler, TX 75708-3154, USA; tsuyoshi.sakai@uthct.edu (T.S.); yungyeon.choo@uthct.edu (Y.C.); osamu.sato@uthct.edu (O.S.); reiko.ikebe@uthct.edu (R.I.); ann.jeffers@uthct.edu (A.J.); steven.idell@uthct.edu (S.I.); torry.tucker@uthct.edu (T.T.)

* Correspondence: Mitsuo.Ikebe@uthct.edu; Tel: +1-(903)-877-7785; Fax: +1-(903)-877-5438

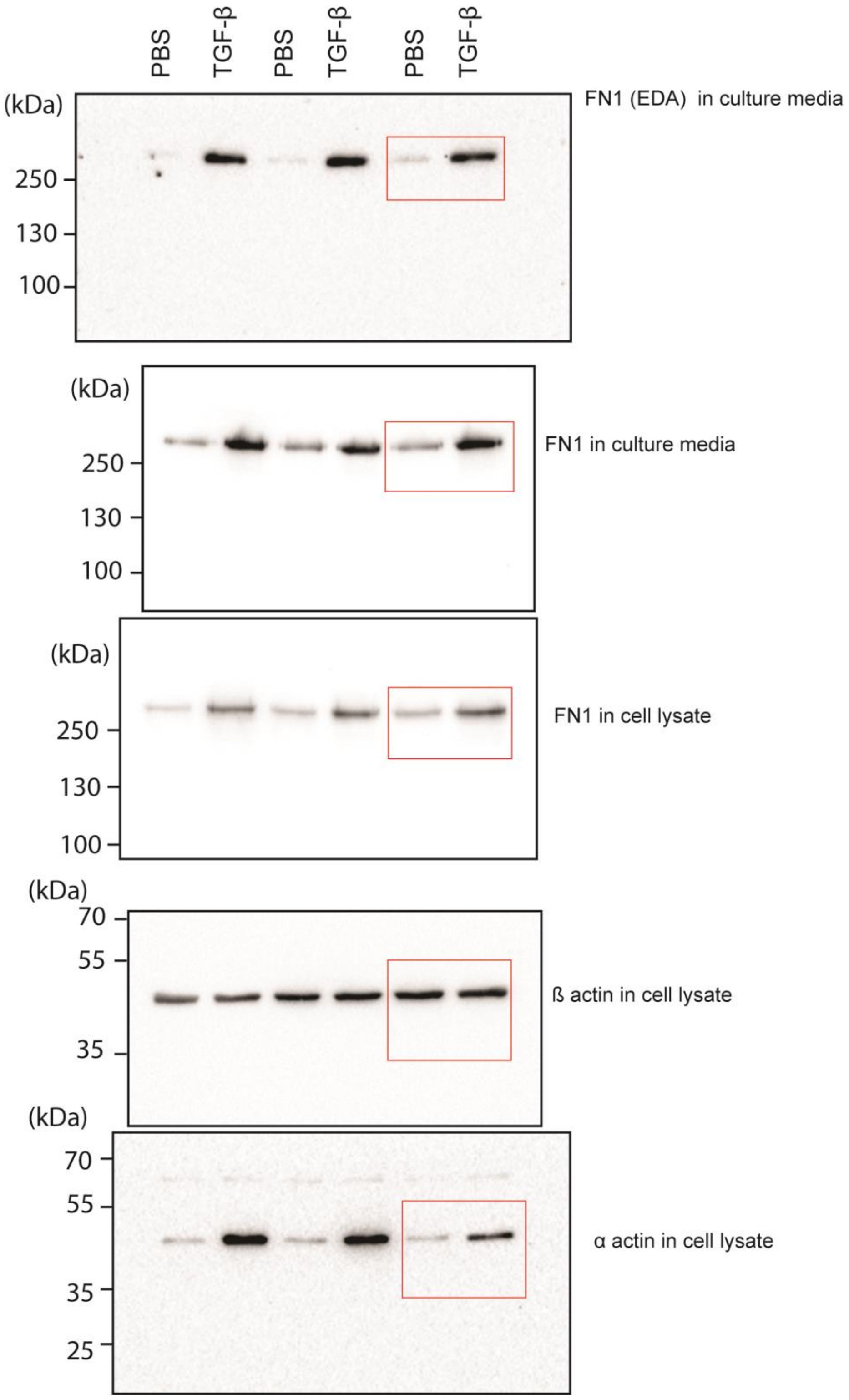

Supplementary Figure S1. Full-length blots for Figure 2B.

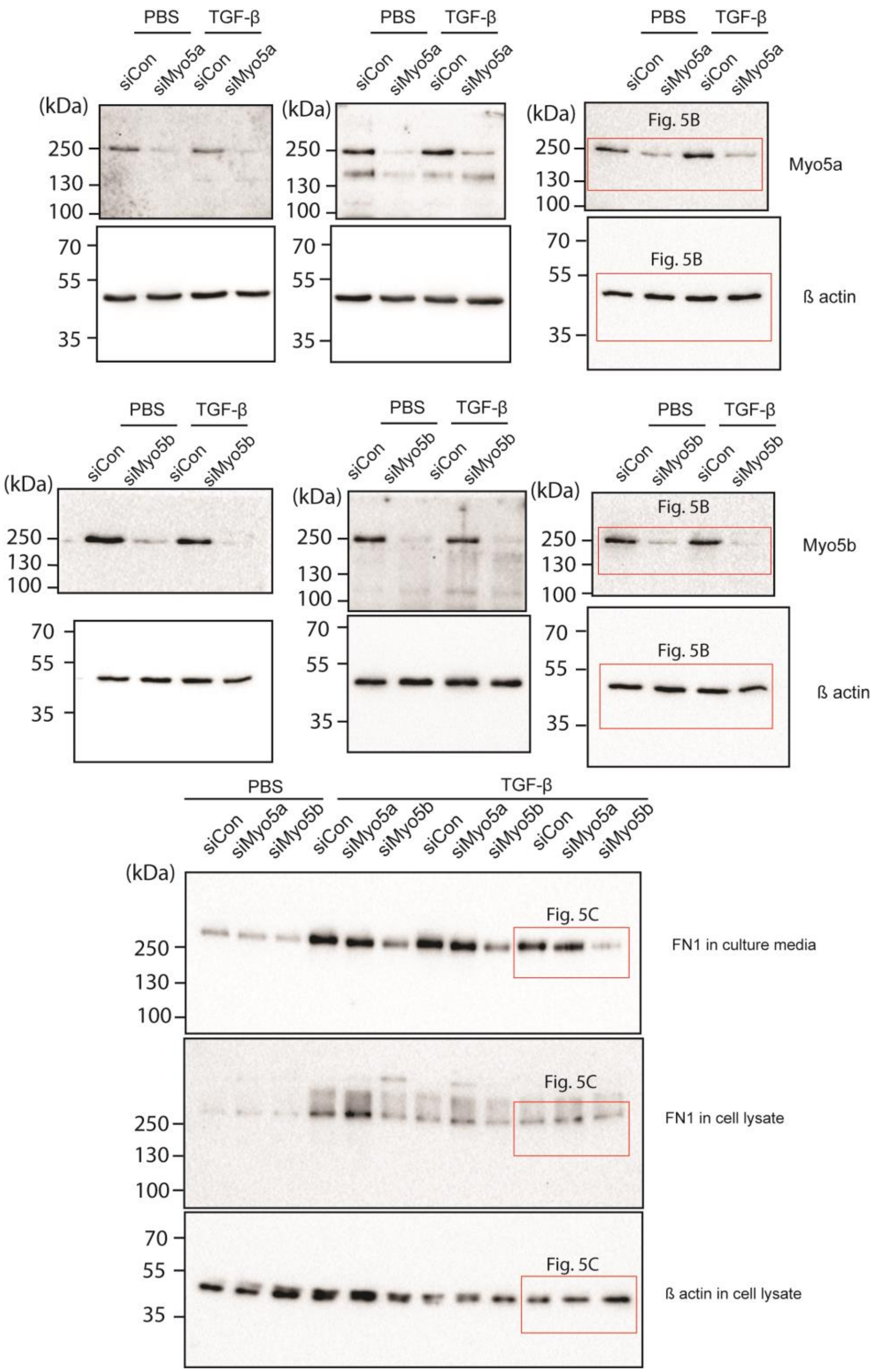

Supplementary Figure S2. Full-length blots for Figure 5.

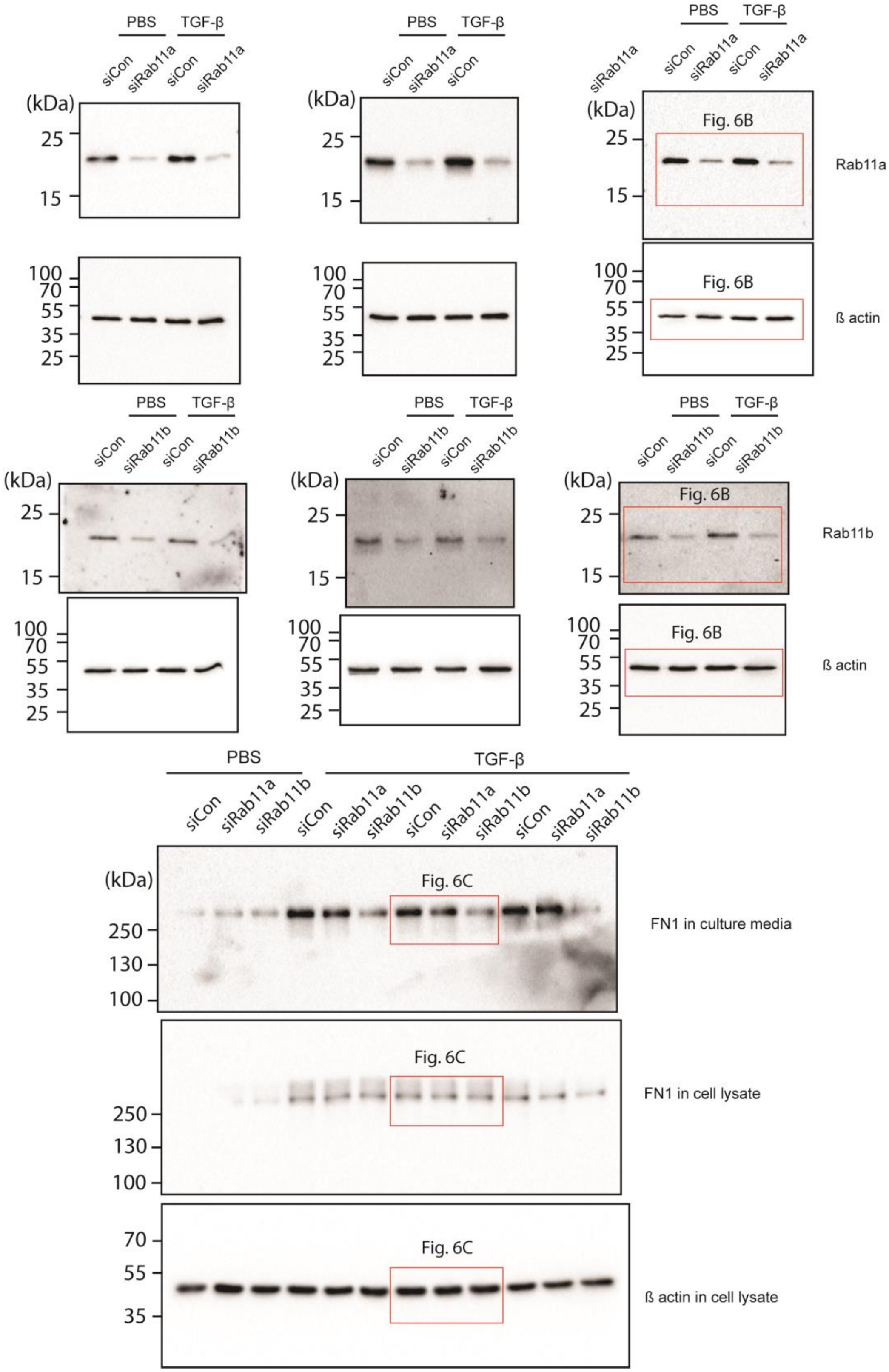

Supplementary Figure S3. Full-length blots for Figure 6.

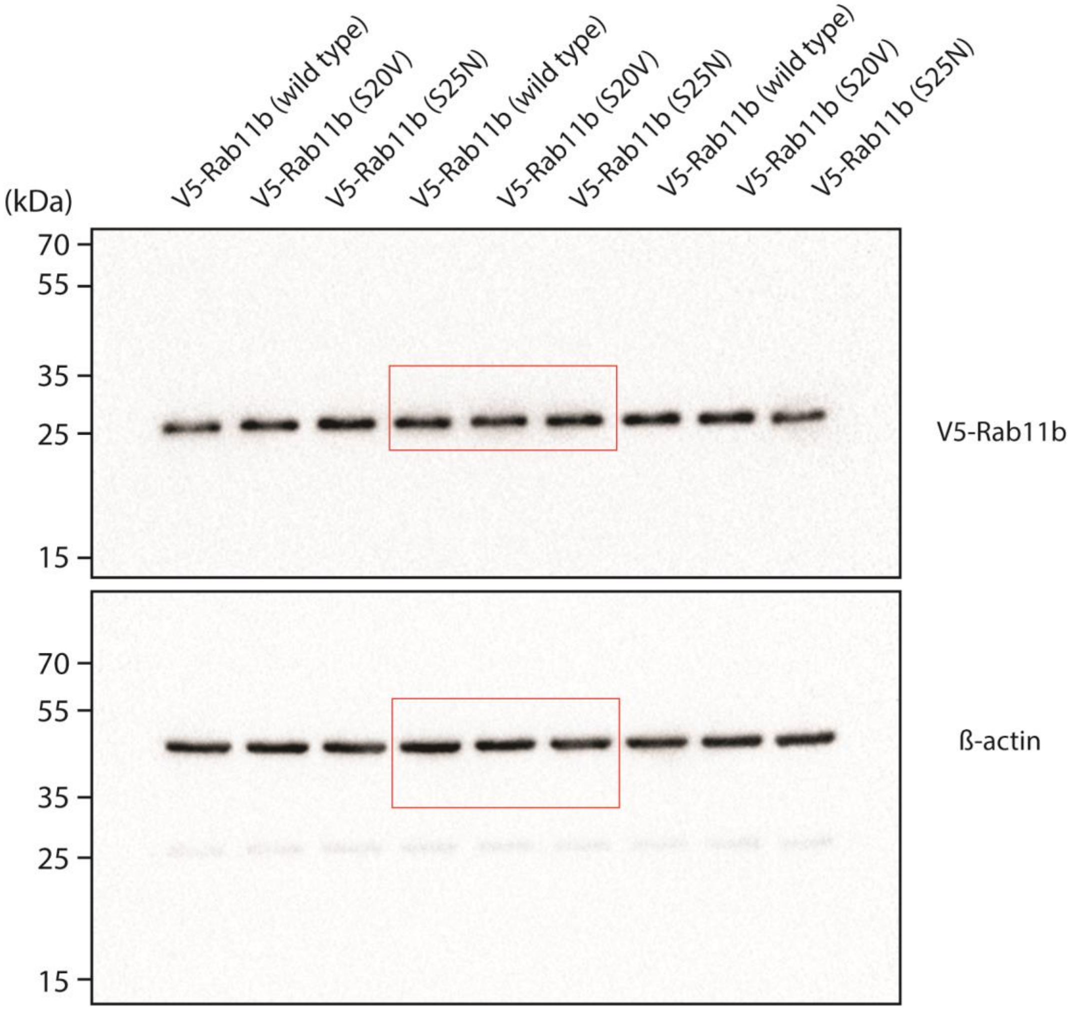

Supplementary Figure S4. Full-length blots for Figure 10.

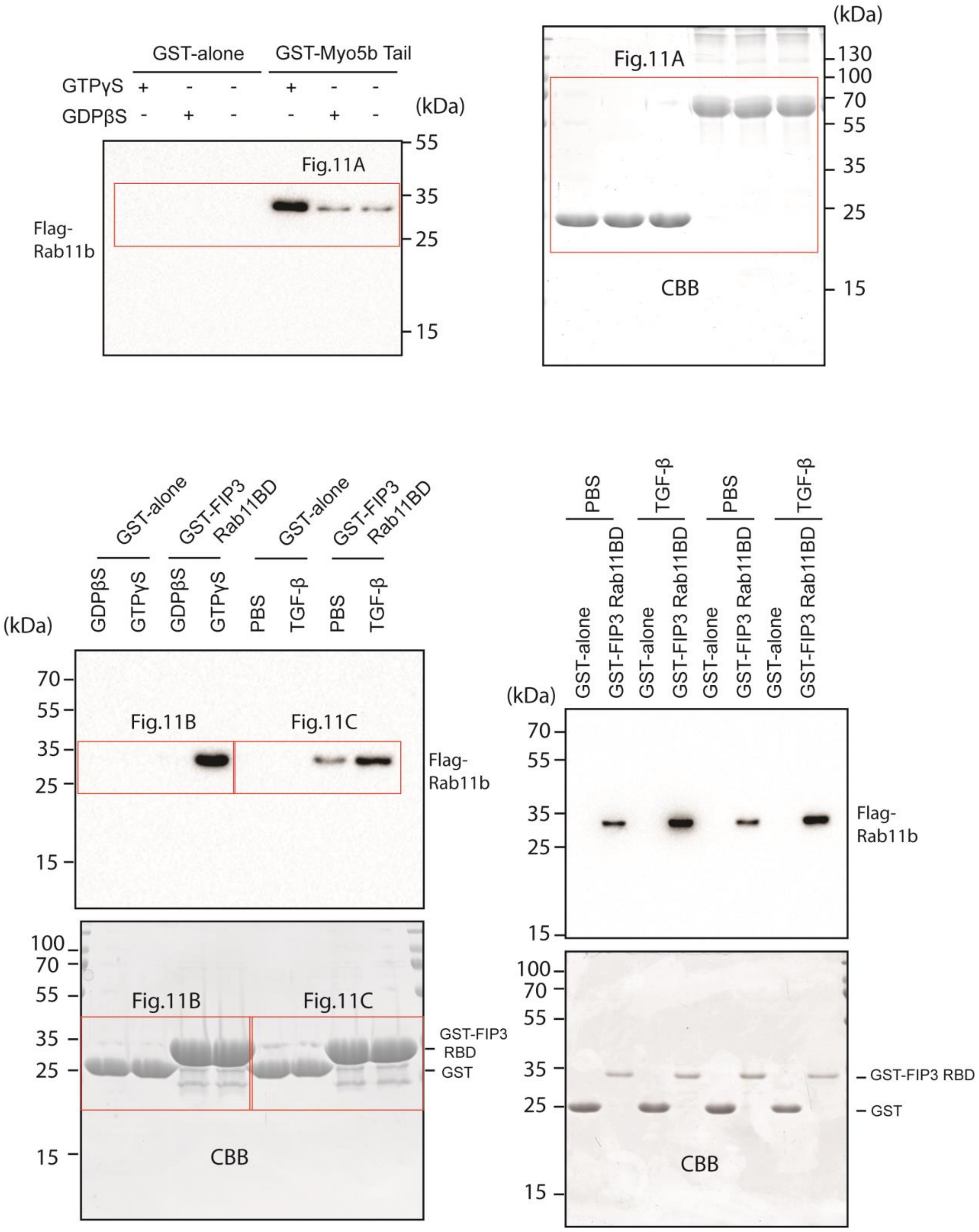

Supplementary Figure S5. Full-length blots for Figure 11.

Supplementary Video S1. Live imaging of Myo5b and FN1 in HPMCs. The GFP-Myo5b and mCherry FN1(EDA) expressing cell was imaged using DeltaVision OMX.
